# Inhibition of neprilysin with sacubitril without RAS blockage aggravates renal disease in Dahl SS rats

**DOI:** 10.1080/0886022X.2021.1879856

**Published:** 2021-02-04

**Authors:** Iuliia Polina, Morgan J. Spicer, Mark Domondon, Ryan S. Schibalski, Elizaveta Sarsenova, Regina F. Sultanova, Daria V. Ilatovskaya

**Affiliations:** a Department of Medicine, Division of Nephrology, Medical University of South Carolina, Charleston, SC, USA; b Saint-Petersburg State Chemical Pharmaceutical University, St. Petersburg, Russia

**Keywords:** Sacubitril, hypertension, neprilysin, kidney, salt-sensitivity

## Abstract

Salt-sensitive (SS) hypertension is accompanied with severe cardiorenal complications. In this condition, elevated blood pressure (BP) resulting from salt retention is associated with counterintuitively lower levels of atrial natriuretic peptide (ANP). In plasma, ANP is degraded by the neprilysin; therefore, pharmacological inhibition of this metalloprotease (i.e., with sacubitril) can be employed to increase ANP level. We have shown earlier that sacubitril in combination with valsartan (75 μg/day each) had beneficial effects on renal function in Dahl SS rats. The goal of this study was to evaluate the effects of a higher dose of sacubitril on renal damage in this model. To induce hypertension, male Dahl SS rats were fed a 4% NaCl diet (HS) for 21 days, and were administered sacubitril (125 μg/day) or vehicle *via* s.c. osmotic pumps. At the end of the HS challenge, both groups exhibited similar outcomes for GFR, heart weight, plasma electrolytes, BUN, and creatinine. Sacubitril exacerbated kidney hypertrophy, but did not affect levels of renal fibrosis. We also observed aggravated glomerular lesions and increased formation of protein casts in the sacubitril-treated animals compared to controls. Thus, in Dahl SS rats, administration of sacubitril without renin-angiotensin-system blockage had adverse effects on renal disease progression, particularly in regards to glomerular damage and protein cast formation. We can speculate that while ANP levels are increased because of neprilysin inhibition, there are off-target effects of sacubitril, which are detrimental to renal function in the SS hypertensive state.

## Introduction

More than 46% of the US population suffers from high blood pressure (BP) [1]. Genetic predisposition to hypertension [1–4], along with various lifestyle and dietary habits, such as high daily salt intake, can significantly increase the risk of mortality, especially in ‘salt sensitive’ (SS) individuals [2,5–9]. A sustained high salt diet provokes hypertension and severe renal damage in the SS population, coupled with cardiovascular complications [2,10–12]. Clinical studies have shown that in response to high salt intake, when compared to salt-resistant individuals, SS patients manifest abnormal renal hemodynamics including a substantial decrease in renal plasma flow, an increase in filtration fraction, and elevated intraglomerular pressure [11,13]. Decline in renal function of SS hypertensive patients is a significant risk factor in the progression to end-stage renal disease (ESRD) and kidney failure. Treatments preventing or attenuating renal damage in SS individuals may improve long-term cardiorenal outcomes for those affected.

Physiological mechanisms underlying the elevation of BP in SS hypertension include abnormalities in natriuretic and renin-angiotensin (RAS) systems [2,4,9,14,15]. Evidence suggests that the RAS of SS individuals is suppressed [4,14,16,17]. Low level of plasma renin, an indicator of RAS suppression, was reported in Dahl SS rats, an animal model of SS hypertension [9,15,18–21]. The natriuretic peptide system is considered a hormonal counteractant to RAS: in this system, atrial natriuretic peptide (ANP) is a key player, as it helps maintain the water-electrolyte balance in the kidneys by acting as a direct vasodilator, increasing the glomerular filtration in concurrence with promotion of diuresis and natriuresis [22–24]. Besides hemodynamic impacts, ANP possesses antihypertrophic and antifibrotic effects in the kidney and myocardium [23,25,26]. ANP is mainly produced by the heart in response to cardiac wall stretching by volume overload. In hypertension, ANP function could be compromised. Clinical studies have demonstrated decreased levels of circulating ANP in hypertensive patients [4,27,28]. In animal models, disruption of *Nppa* led to arterial BP elevation [29,30]. Genetic studies in mice with *Npr1* deficiency (*Npr1* encodes natriuretic peptide receptor – A), resulted in fibrosis, hypertrophic growth and remodeling of the kidney tissue, massive albuminuria, and podocyte injury, along with elevated BP and cardiac hypertrophy, which are observed in human hypertensive individuals [24,25,30,31]. Given that ANP plays an important regulatory role in renal and cardiovascular hemodynamics, the strategy to increase its circulating level is very appealing.

One way of increasing circulating ANP level is to inhibit its degradation. Neprilysin (NEP) is a neutral endopeptidase responsible for degradation of a variety of bioactive peptides, including ANP [22,23,26,32,33]. This enzyme is extensively expressed in the kidneys, particularly in the renal proximal tubule [26,34]. The primary rationale for NEP inhibition as therapeutic agent was a possibility to increase the endogenous levels of natriuretic peptides (NPs), especially in patients with cardiovascular diseases (CVD). However, it is clear now that many of these inhibitors have more than one target. In the 1990s, several studies revealed potential beneficial effects of NEP inhibitor (NEPi) monotherapy in patients with heart failure and essential hypertension [35–38]. Indeed, patients treated with the NEPi candoxatril manifested significant increases in ANP level, although, there was no clinically relevant reduction in BP [35]. Mild effects on BP were demonstrated with a NEPi sinorphan [37].

Recently, the NEPi sacubitril was approved for chronic administration in heart failure, in combination with valsartan, an angiotensin receptor blocker (ARB) [32,33]. The combination of NEPi and RAS blockade has been researched as a treatment option for diabetes-related cardiorenal pathologies. A 2016 study that demonstrated a significant reduction in heart failure hospitalizations in diabetic patients treated with sacubitril/valsartan also showed it to be a safe and sustainable treatment for a subset of patients with ESRD [39,40]. More recently, an analysis of the PARADIGM-HF trial examined changes in estimated GFR (eGFR) in patients with and without diabetes [41]. This study found that patients treated with sacubitril/valsartan had a slower rate of decline in eGFR, and that diabetic patients had a more drastic attenuation of eGFR decline than patients without diabetes [41]. In a follow-up review to the PARADIGM-HF study, it was suggested that because patients with type 2 diabetes display a decreased response to endogenous NPs, neprilysin has a higher detrimental effect, and therefore NEPis can be more beneficial for renal health [42]. Overall, there has been little research done into the efficacy of sacubitril in regard to renal as opposed to cardiac health; however, this is a promising line of research.

The current study focused on the effects of NEPi monotherapy on renal function of SS hypertension. We initially hypothesized, based on our prior data [43], that an increase in sacubitril dosage (from 75 μg/day used previously, to 125 μg/day) would enhance the mild beneficial effects of the drug that we observed at a lower dose. The rationale for testing sacubitril monotherapy stemmed from the fact that the RAS is suppressed in the Dahl SS rat [18,19]; based on this, we speculated that a combination therapy with an ARB might not be required for the SS condition. Contrary to our expectations, sacubitril monotherapy resulted in exacerbation of the renal lesions in the Dahl SS rat, likely due to the off-target effects of this NEPi, which manifested at a higher dose.

## Methods

### Experimental protocol and animals

**Figure 1.**
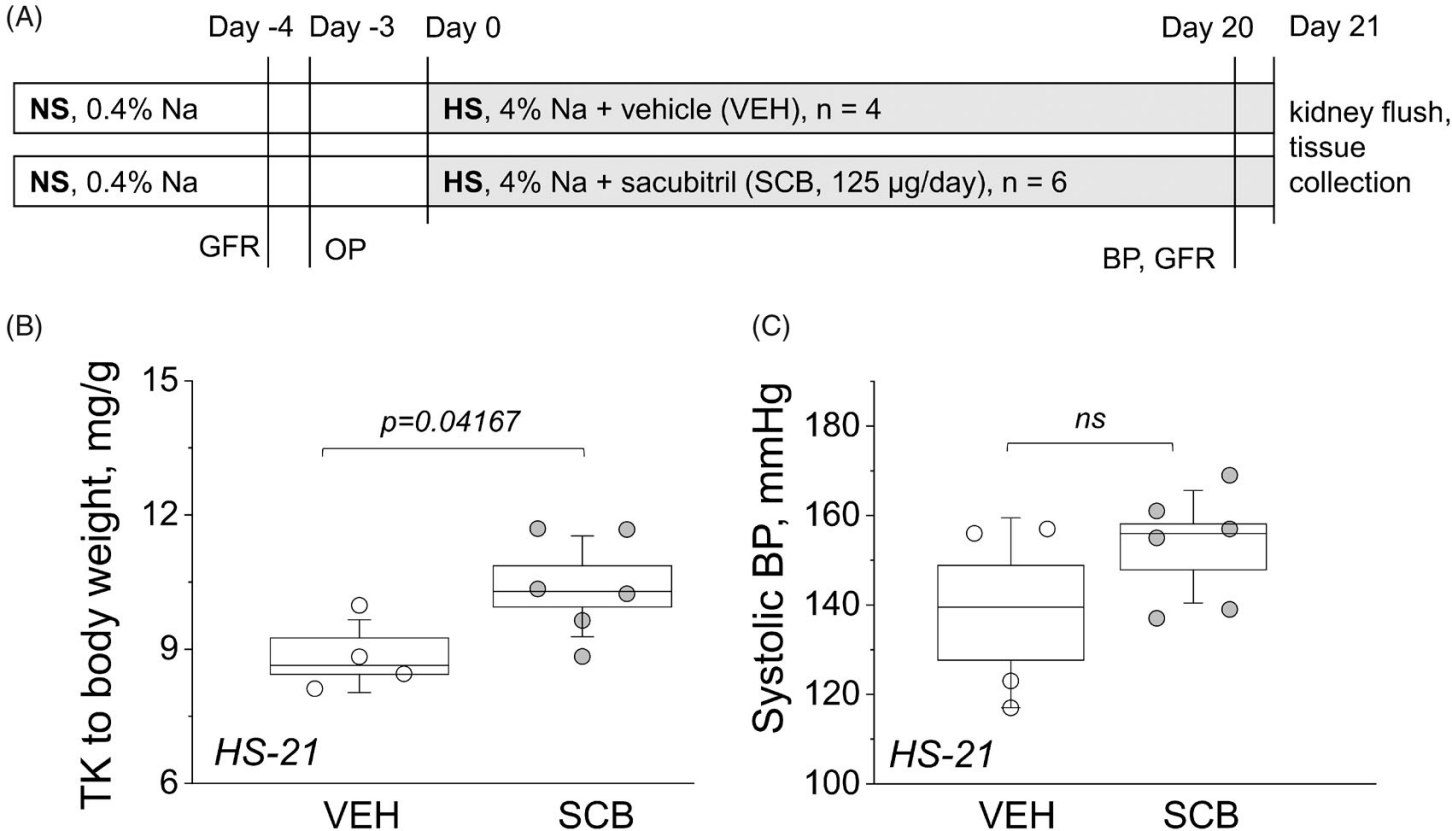
Experimental protocol and basic endpoint parameters. (A) A schematic representation of the experimental protocol. Dahl SS rats were allowed to acclimate for one week on a 0.4% NaCl diet before being swapped to a HS diet for 21 days. GFR was measured, OPs were installed before the HS challenge, and then GFR and BP were measured on day 20. Animals were euthanized and a kidney flush procedure was performed on day 21; kidneys, hearts, and blood plasma were harvested. NS, normal salt diet; HS, high salt diet; GFR, glomerular filtration rate measurements; BP, blood pressure measurement; OP, osmotic pump. (B) Endpoint kidney-to-total body weight ratios. (C) Endpoint systolic BP. One-way ANOVA was used for statistical analysis. P values are shown for comparisons where *p* < .05.

All experimental procedures were approved by the Institutional Animal Care and Use Committee (IACUC) of the Medical University of South Carolina and performed in accordance with the NIH guidelines for Care and Use of Laboratory Animals. Seven-week-old male Dahl SS rats (Charles River Laboratories, USA) were kept on a standard day-night cycle, and had *ad libitum* access to water and food during the experimental protocol. Purified AIN-76A-based rodent diets manufactured by Dyets Inc have been used for all experiments. For one week upon arrival animals were fed a normal (0.4%, NS) NaCl diet. Afterwards, rats were randomly separated in two groups treated with sacubitril at 125 µg/day, or vehicle as a control, for 21 days beginning at 8 weeks of age, *via* subcutaneously implanted osmotic pumps (Alzet 2ML4, 2.5 µL/hour). 3 days after the pump implantation, animals were switched to a high (4%, HS) NaCl diet for 21 days. Body weight, tail cuff blood pressure, and glomerular filtration rate (GFR) measurements were obtained the day before a surgery and at day 20 of HS diet. At the end of the experimental protocol (day 21), animals were euthanized, followed by a kidney flush procedure with blood and tissue collection. The schematic of the experimental protocol is depicted in Figure 1(A).

### Tail cuff blood pressure measurements

Systolic BP was measured in conscious rats by tail-cuff plethysmography (IITC Life Science Inc., USA). Baseline measurements were taken the day before osmotic pump installation, and the end-point BP data were obtained on day 20. Animals were trained for 1 week before beginning BP measurements to minimize the impact of stress on the experimental outcomes. Briefly, animals were encouraged to walk into the restrainer, and the front holder was carefully adjusted to prevent excessive movements. Afterwards the occlusion cuff with the sensor was optimally positioned close to the base of the rat’s tail. The occlusion cuff was inflated to approximately 220 mmHg and deflated to 50 mmHg. Each animal had 5 recording cycles, and results were presented as the mean value.

### Glomerular filtration rate measurements

To evaluate GFR, we measured fluorescent FITC-labeled inulin (TdB Consultancy AB, Uppsala, Sweden) plasma kinetics following an intravenous administration. The high-throughput technique was slightly modified from the protocol for mice [44] and has been described previously [45]. In brief, animals were weighed and subsequently briefly anesthetized *via* isoflurane inhalation. Afterwards, pre-dialyzed 20 mg/mL of FITC-inulin solution in 0.9% saline was slowly injected *via* the lateral tail vein from either side at 2 µL/g of body weight, and animals were allowed to regain consciousness. 10 µL of blood was collected by tail bleed into Na-Heparin capillaries (Hirschmann Laborgerate, Eberstadt, Germany) at 3, 5, 8, 16, 25, 40, 60, 80, 100 and 120 min post injection. After centrifugation, plasma was diluted 1:10 with 0.5 mol/L solution of HEPES, pH 7.4 followed by FITC fluorescence intensity measurements using a NanoDrop 3300 Fluorospectrometer (Thermo Fisher Scientific, Wilmington, DE, USA). Inulin concentration was plotted versus time and fitted with bi-exponential decay function in the OriginPro 9.0 program (OriginLab, Northampton, USA). To calculate GFR, we used a two-compartment clearance model described by the following equation GFR = n/(A/K1 + B/K^2^), where n is amount of inulin delivered by injection; A and B are the Y-intercept values of the two decay rates, and K1 and K2 are the decay constants for the distribution and elimination phases, respectively.

### Kidney flush and tissue collections

To clear the blood from the kidneys, a kidney flush surgery was performed, as described previously [46,47]. Briefly, the animals were anesthetized *via* isoflurane inhalation, and then placed on the heated (37 °C) surgical platform. Next, the abdominal cavity was opened to expose the kidneys and provide access to the abdominal aorta in order to place the catheter and collect blood into heparin-containing centrifuge tubes. After the blood collection, the kidneys were perfused with PBS/heparin solution at 2 mL/min, tissues were then harvested, weighed, and fixed in 10% neutral buffered formalin (Fisher Sci., USA) for further histomorphometric evaluations.

### Histology

After 24 h, the 10% neutral buffered formalin containing the kidneys was substituted with 70% ethanol (Fisher Sci., USA), and the tissue was routinely processed for histological analysis. Paraffin-embedded kidney and heart tissues were sectioned and further stained with Masson Trichrome in order to analyze the degree of tissue fibrosis and glomerular injury. Histological analysis was performed in a blinded manner.

### Imaging and analysis

For renal damage evaluation we used the method described previously [47]. Briefly, the glomerular injury analysis was performed in Masson Trichrome stained slides using a Nikon Plan Fluor optical lens of 20X magnification on a Nikon Eclipse Ti-2 microscope. Glomeruli were scored on a 0–4 scale, where 0 is a healthy glomerulus with no sclerosis. The score 1 shows a 1–25% of mesangial expansion and sclerosis, 2 represents 26–50% of mesangial expansion and sclerosis, glomeruli with a 51–75% of mesangial expansion and sclerosis had a score 3, and a score of 4 was given to a 76–100% mesangial expansion and sclerosis in glomeruli. To evaluate the fibrosis and protein casts, Masson Trichrome stained tissue slides were scanned with a Perkin Elmer Vectra Polaris Automated Quantitative Pathology Imaging System slide scanner, followed by the digital analysis or scoring. For protein casts, a 1–4 scale was used for assessments of protein casts formation in kidney tissue. In a 4x image, a score of 1 characterized a tissue where 0–10% protein casts were detected. 10–20% of detected protein casts in kidney tissue was characterized by a score of 2. Scores 3 and 4 were given to kidney tissue samples with detected 20–30% and more than 40% protein casts, respectively. FIJI software (NIH) was utilized for quantification of fibrosis: the region of interest (ROI, entire cortex and medulla) was selected and the area of the ROI was measured. Then, the Color Convolution Plugin in the ImageJ program allowed us to calculate the percentage of total fibrotic area using a threshold tool. All representative images were acquired at 10X using a Nikon Ti-2 microscope equipped with NIS Elements software and DS-Fi3 camera.

#### BUN and creatinine

Plasma electrolytes were measured with the CareLyte electrolyte analyzer (Diamond Diagnostic Inc., USA) immediately after collection of blood. Remaining plasma was snap-frozen in liquid nitrogen for further analysis. Plasma creatinine levels were measured using the Quantichrom Creatinine Assay Kit (DICT-500, BioAssay Systems). A standard curve was created from the stock 50 mg/dL creatinine standard (6 mg/dL, 2 mg/dL, 1 mg/dL, 0.5 mg/dL and 0 mg/dL). Creatinine concentrations were determined by measuring absorbance per the manufacturer’s instructions. BUN was measured using a urea assay kit (Abnova, #KA1652) according to manufacturers’ instructions.

### Statistical analysis

The data are presented as means ± SEM and indicated along with n values in each figure legend. All box plots are representing SEM values; the whiskers being the SD, and the central line showing the mean or median value. Statistical tests used to estimate the differences between the two groups are denoted in each Figure legend. Values of *p* < .05 were defined to be statistically significant. OriginPro 9.0 software (OriginLab, Northhampton, USA) was used for analysis.

## Results

### Assessment of basic renophysiological parameters following drug administration

Figure 1(A) shows the timeline of the experimental protocol (described in detail in METHODS). Body weight was measured in both groups before the start of the HS challenge (on the NS diet) and after 21 days on the HS diet. Here, we chose to use the 125 μg/day dose of sacubitril (compared to 75 μg/day used by us previously [43], an average of ∼0.4 mg/kg/day was administered to rats throughout the protocol. For human patients, recommended starting oral dose of a combination drug (Entresto^®^) is 25–50 mg/twice a day (0.7–1.5 mg/kg/day for a 70 kg person). Kidney-to-body-weight ratio was significantly higher in the sacubitril-treated (SCB) group at 10.4 mg/g versus the vehicle-only control (VEH) group at 8.8 mg/g, with *p* < .05 (Figure 1(B)). Figure 1(C) displays the 21-day endpoint systolic blood pressure (BP) and shows a trend for BP increase in the SCB group. Endpoint plasma concentrations of electrolytes Na, K, and Cl were similar between groups, as were heart weights, plasma creatinine, and BUN, as shown in Table 1.

**Table 1.** Endpoint plasma electrolytes, BUN, creatinine and tissue weights. Student's paired t-test was used for significance comparisons.

	VEH	SCB	*p*-value	N
Plasma [Na], mmol/L	149.7 ± 2.02	153 ± 3.3	.52	3, 6
Plasma [K], mmol/L	3.8 ± 0.17	3.27 ± 0.28	.25	3, 6
Plasma [Cl] mmol/L	112 ± 0.01	113 ± 3.68	.86	3, 6
Body weight (NS), g	224.4 ± 5.2	239.7 ± 5.3	.31	4, 6
Body weight (HS), g	325.3 ± 7.4	339.8 ± 9.7	.43	4, 6
Heart Weight	1.28 ± 0.08	1.40 ± 0.09	.31	4, 6
Left Kidney Weight, g	1.49 ± 0.10	1.81 ± 0.07	.03	4, 6
Right Kidney Weight, g	1.38 ± 0.90	1.71 ± 0.04	.004	4, 6
Plasma creatinine, mg/dL	0.49 ± 0.04	0.50 ± 0.04	.89	3, 6
BUN, mg/dL	1.28 ± 0.18	1.29 ± 0.17	.99	3, 6

Data is presented as mean value ± the standard error of the mean (SEM). The N-column represents the number of rats used for analysis in each group, and is listed as (VEH, SCB).

### Assessment of cardiac tissue following drug administration

**Figure 2.**
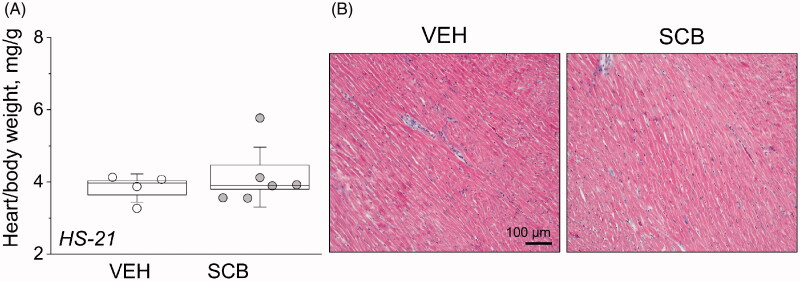
Sacubitril’s effects on the heart. (A) Endpoint heart-to-total body weight ratios. (B) Histological characterization of cardiac tissue using Masson trichrome staining. Representative images of cardiac tissues from experimental rats isolated at the end point of the experimental protocol [high-salt diet, upon administration of vehicle (VEH) or sacubitril (SCB)]. One-way ANOVA was used for statistical analysis. *p* values are shown for comparisons where *p* < .05.

At the end of the 21-day HS challenge, hearts were collected and weighed. As indicated in Figure 2(A), heart-to-body-weight ratios were similar between VEH and SCB groups. Representative images of cardiac histology are shown in Figure 2(B), where Masson Trichrome staining shows no notable difference in cell morphology or cardiac fibrosis.

### Renal damage

**Figure 3.**
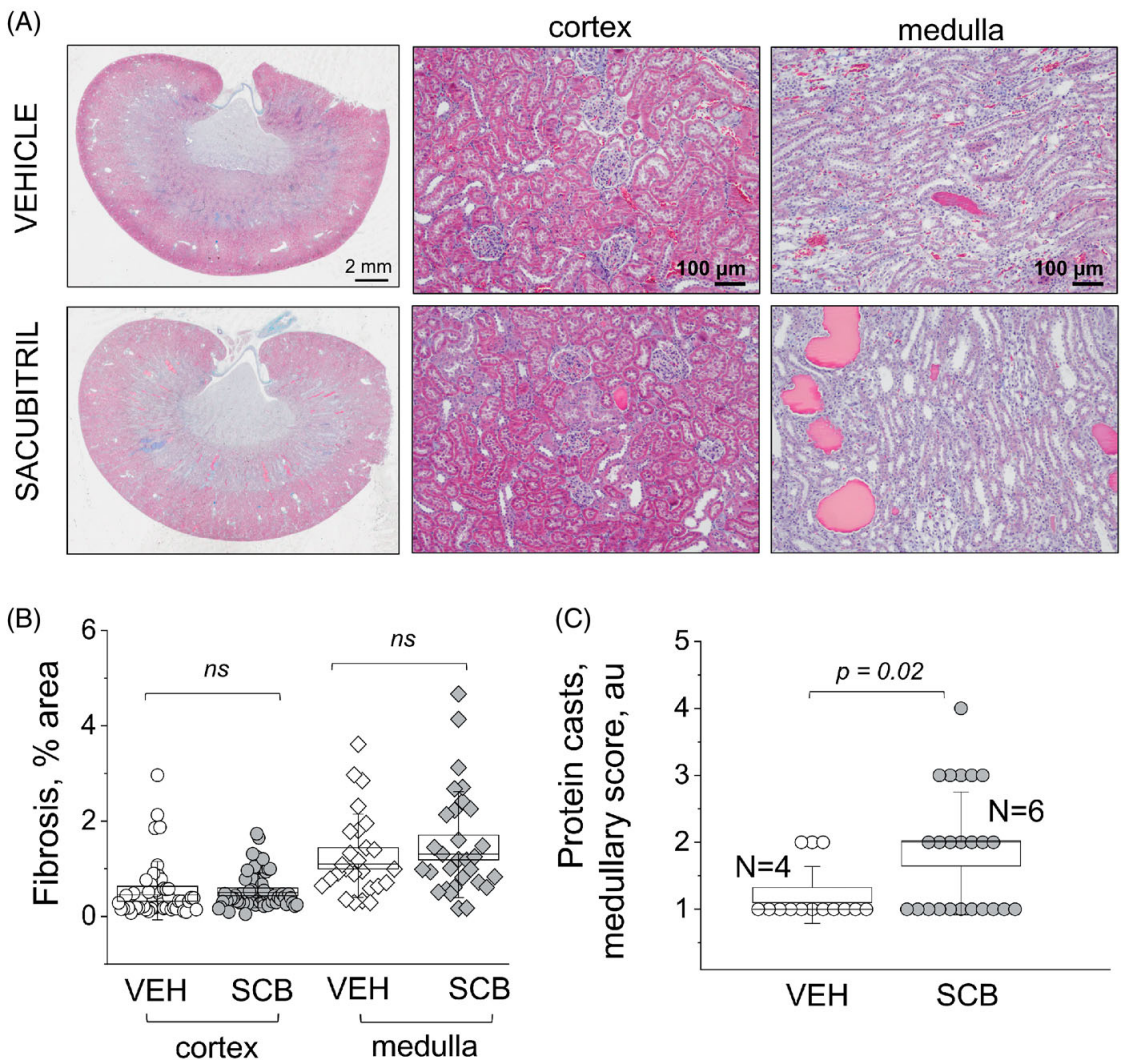
Histological characterization of renal damage with Masson trichrome staining. (A) Representative images of cortical and medullary renal tissues from experimental rats isolated at the end point of the experimental protocol [high-salt diet, upon administration of vehicle (VEH) or sacubitril (SCB)]. The first column shows scans of coronal midsections of kidneys stained with Masson trichrome; fibrotic tissue appears blue. The second and third columns demonstrate representative images taken in the cortexes and medulla, respectively. (B and C) graphs summarizing the analysis of percentage of fibrosis (*B*) and protein cast scoring (*C*). a.u., arbitrary units. Two-way ANOVA with Tukey *post-hoc* was used for significance comparisons. Each point on the graphs denotes data obtained from one image, with 16 images being taken per kidney: 10 from each cortex, and 6 from each medulla. *p* values are shown for comparisons where *p* < .05.

**Figure 4.**
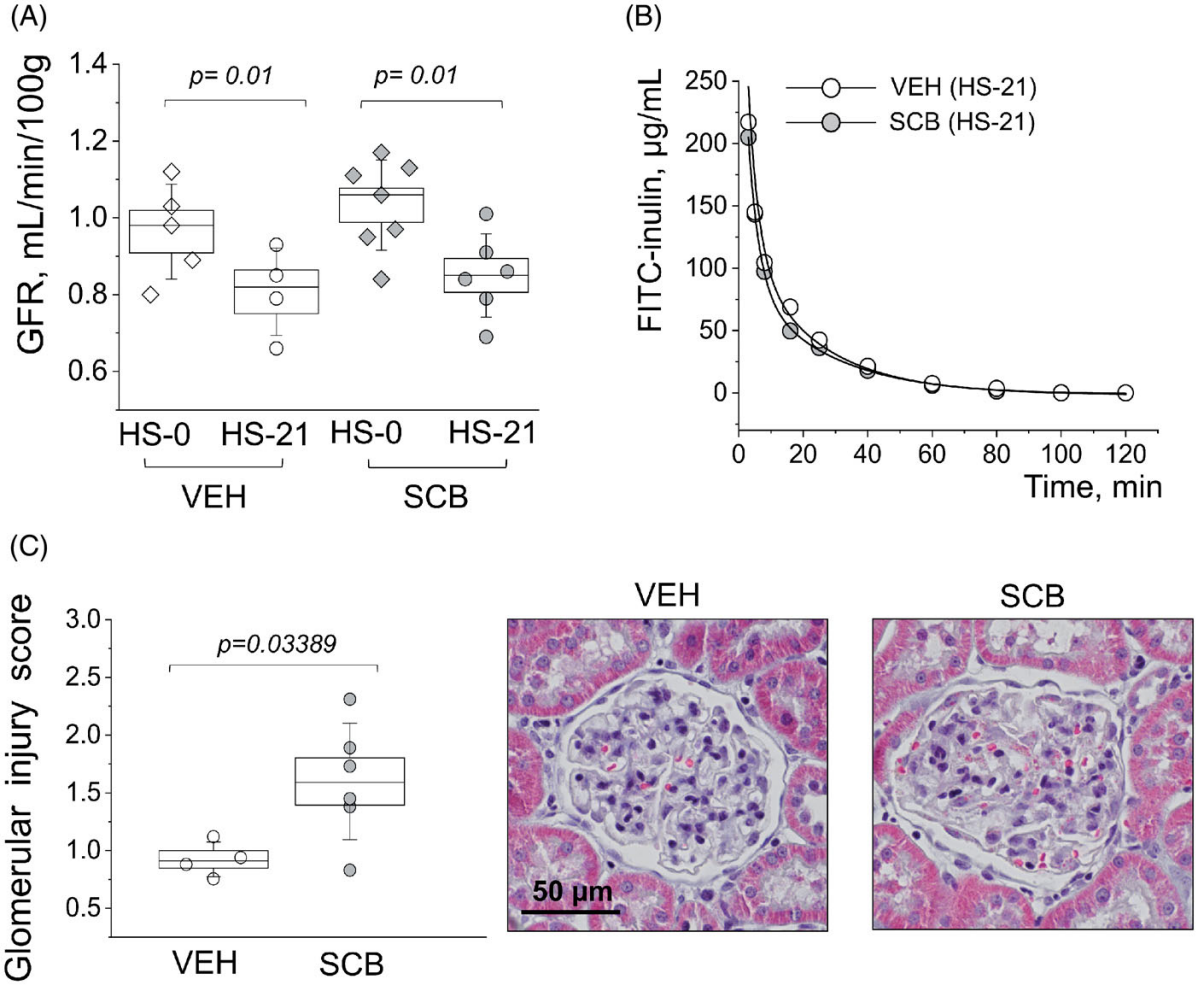
Characterizations of glomerular function and damage. (A) Start vs endpoint glomerular filtration rates (GFR). (B) Representative FITC-inulin clearance curves from conscious freely moving rats injected with FITC-inulin. (C) Glomerular injury as determined by scoring individual glomeruli for damage; each point is an average of 100 randomly scored glomeruli per kidney. Images are representatives of glomeruli from Masson trichrome stained tissues, from VEH and SCB animals. Two-way ANOVA with Tukey *post-hoc* was used for significance comparisons. *p* values are shown for comparisons where *p* < .05.

Figure 3(A) shows representative histology between the groups at 2 mm and 100 μm high-resolution scans. Fibrosis is depicted as blue in Masson Trichrome stain, and quantified in Figure 3(B); even though there is no statistical significance between SCB and VEH groups, there is a distinct expected difference between cortical and medullary fibrosis (*p* = 7.8 × 10^−5^ and *p* = 3.0 × 10^−8^ for the control and treated groups, respectively) [48]. Figure 3(C) shows scores representative of protein casts in the medulla. Protein cast formation was significantly higher (*p* = .02) in the SCB group at a score of 1.8, versus vehicle-treated animals which scored 1.2. The average glomerular filtration rate (GFR) was lower (from 1.0 to 0.83) after the HS challenge; however, there was no difference between VEH and SCB groups (Figure 4(A)). Representative FITC-inulin elimination curves for day 21 are shown in Figure 4(B). Interestingly, the glomerular injury score was significantly higher in the sacubitril-treated group than in the vehicle-treated control with a *p* < .05, as shown in Figure 4(C). Representative histological images also depict expansion of mesangial matrix in glomerulus.

## Discussion

This study utilized Dahl SS rats as an established model for SS hypertension and renal disease; this strain is known to demonstrate phenotypes and characteristics of the renal disease typical to the SS human population [49–52]. Administration of sacubitril during the 21-day HS challenge resulted in significant kidney hypertrophy and glomerular damage, as well as increased formation of protein casts in the renal medulla. In our hands, cardiac hypertrophy (heart to body weight ratio) was similar between the vehicle and sacubitril-treated groups. Dahl SS rats do not typically exhibit profound heart disease [49,53,54]. Furthermore, this model is known to be deficient in renin and prorenin, which leads to suppression of the RAS [18,19]. Over-activation of RAS is implicated in both heart failure and in cardiac fibrosis, and Dahl SS rats have relatively low incidence rates of both [55,56]. In combination with valsartan, however, sacubitril is known to attenuate cardiac hypertrophy and fibrosis [57]. Therefore, the effects of sacubitril on cardiac function and fibrosis in SS hypertension remain to be further studied, especially in SS models with more pronounced heart damage or more advanced heart disease.

In humans, NEPis have been historically studied as a treatment of heart failure. Intravenous administration of candoxatril was shown to reduce pulmonary capillary pressure and right atrial pressure in patients with heart failure [58]. Similar effects were seen with the NEPi ecodatril [59], but neither drug finished development and reached the market because of their inability to cause a long-term, sustained reduction in BP. In our study, though BP was not significantly higher, we observed exacerbated renal hypertrophy in the SCB group, which commonly accompanies hypertension [60,61], and is caused in part by microvascular barotrauma [62]. In our previous study [43] with a lower dose of sacubitril, we reported no changes in kidney to body weight ratio in the SCB group. We can speculate that when sacubitril dosage is increased, in the absence of ARBs, sacubitril treatment results in increased RAS activation due to the side effects of neprilysin inhibition [33]; however, more extensive mechanistic studies are required to make definite conclusions.

Despite differences in sacubitril dosing between this and our previous study, there are some similarities. When comparing protein cast formation with proteinuria-related data from our prior work [43], it is apparent that sacubitril alone does not attenuate protein casting at either dose. Concerning glomerular damage, we observed a significantly higher glomerular injury score (1.6 a.u.) in the SCB treated animals compared to control (0.92 a.u.). However, this difference in glomerular injury was not reflected in GFR: the HS diet caused a significant GFR decrease in both the SCB and VEH group when compared to the GFR before the HS challenge, while the endpoint values for SCB and VEH groups remained similar. Renal blood flow depends on a variety of factors, and glomerular fibrosis assessed in the scoring protocol may not fully reflect the functional ramifications of SCB administration. *In vivo* renal blood flow measurements in VEH and SCB treated animals would be helpful to reconcile these findings.

Interestingly, we found in our prior study that attenuation of renal medullary fibrosis, proteinuria, and protein cast formation were largely driven by the combination of sacubitril with the angiotensin receptor blocker (ARB) valsartan, as opposed to sacubitril alone. This is in line with other studies, where it has been demonstrated that valsartan is able to partially attenuate fibrosis in diabetic nephropathy [63] and alleviate renal injury in CVD patients [64]. Also notable from our prior study was the reduction in systolic BP in both sacubitril/valsartan and valsartan only groups, which contrasts with the trend for the increase demonstrated by sacubitril only dosage in this study. Despite dosage differences, the trend for higher BP in sacubitril-only treated animals persists in both this and the prior study. Our findings highlight the fact that in order to achieve attenuation of renal damage and hypertension, combination of a NEP inhibitor like sacubitril with an ARB such as valsartan is indeed needed.

It is important to mention that NEP has the ability to degrade peptides other than ANP; these include bradykinin, angiotensin II (ANG II), and endothelin 1 [26,32,33,65]. We believe that the off-target effects of NEPi monotherapies significantly contribute to the adverse effects that are observed. Indeed, clinical studies have demonstrated that high doses of NEPis induced systemic vasoconstrictive rather than vasodilatory effects in patients with congestive heart failure (CHF) [38], and potentiated ANG II – induced vasoconstriction effect in healthy volunteers [32,33,66]. Intra-arterial administration of NEP inhibitors (candoxatril or thiorphan) produced vasoconstriction in patients with hypertension, but that effect was alleviated by co-administration of an endothelin 1 receptor blocker [67]. In our prior study [43], we showed that the combination of sacubitril at a lower dose of 75 μg/day with the ARB valsartan is somewhat effective to alleviate the end-organ damage outcomes. In the current study, we can speculate that the inhibition of neprilysin by a higher dose of sacubitril results in more effective accumulation of other neprilysin targets, such as ANG II/bradykinin/endothelin, which overshadow the beneficial effects of ANP level increase. This study utilized a dosage of 125 μg/day of sacubitril, which, without the ameliorative effects of RAS blockade, elicited the damaging effects on the kidney.

It is established now that sacubitril not only suppresses NEP, but also likely increases bradykinin level, thereby increasing the risk for inflammatory processes *via* activation of bradykinin 1 (B_1_) receptors, as well as development of angioedema in animal experimental models [32,33,68]. Therefore, it is becoming increasingly clear that chronic NEP inhibition therapy can have both beneficial and detrimental effects. Here, we demonstrated that in SS hypertension, NEPi without RAS suppression may aggravate renal damage; we speculate that this is due to the off-target effects of NEPi, which need to be taken into account when repurposing NEPis for new diseases.
